# Bis{*N*,*N*,*N*-trimethyl-2-oxo-2-[2-(2,3,4- trihydroxy­benzyl­idene)hydrazin­yl]ethanaminium} tetra­chlorido­zincate(II) methanol solvate^1^

**DOI:** 10.1107/S160053681000615X

**Published:** 2010-02-24

**Authors:** Sladjana B. Novaković, Bojana M. Drašković, Ljiljana S. Vojinović-Ješić, Valerija I. Češljević, Vukadin M. Leovac

**Affiliations:** a ’Vinča’ Institute of Nuclear Sciences, Laboratory of Theoretical Physics and Condensed Matter Physics, PO Box 522, 11001 Belgrade, Serbia; b Department of Chemistry, Faculty of Sciences, University of Novi Sad, Trg Dositeja Obradovića 3, 21000 Novi Sad, Serbia

## Abstract

The asymmetric unit of the title compound, (C_12_H_18_N_3_O_4_)_2_[ZnCl_4_]·CH_3_OH, consists of two Girard reagent-based cations, a tetra­chlorido­zincate anion and a mol­ecule of methanol as solvate. These components are inter­connected in the crystal structure by an extensive network of O—H⋯O, N—H⋯O, C—H⋯O, O—H⋯N, O—H⋯Cl, N—H⋯Cl and C—H⋯Cl hydrogen bonds. The shortest inter­molecular inter­action is realized between the cation and anion [H⋯Cl = 2.29 (5) Å; O—H⋯Cl = 167 (3)°]. C—H⋯O inter­actions also play a important role in the inter­connection of the cations.

## Related literature

For the crystal structures of the related Girard reagent-based ligands and coordination compounds, see: Leovac *et al.* (2006 ▶, 2007 ▶); Vojinović *et al.* (2004 ▶) and references therein; Vojinović-Ješić *et al.* (2008 ▶, 2010 ▶); Revenko *et al.* (2009 ▶). For the crystal structures containing the tetra­chlorido­zincate ion, see: Jin *et al.* (2005 ▶); Valkonen *et al.* (2006 ▶). 
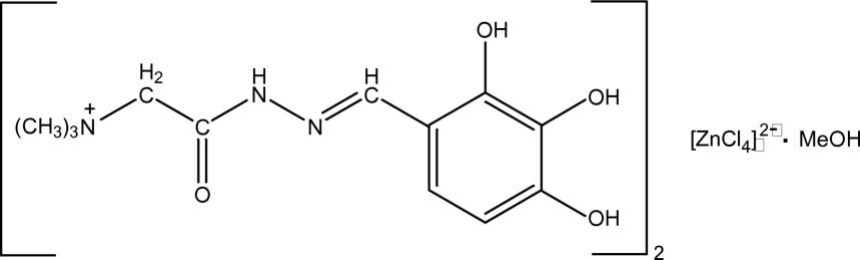


## Experimental

### 

#### Crystal data

(C_12_H_18_N_3_O_4_)_2_[ZnCl_4_]·CH_4_O
*M* *_r_* = 775.82
Triclinic, 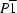

*a* = 9.471 (3) Å
*b* = 13.389 (4) Å
*c* = 14.986 (5) Å
α = 110.90 (4)°
β = 94.91 (4)°
γ = 103.94 (5)°
*V* = 1691.9 (12) Å^3^
*Z* = 2
Mo *K*α radiation
μ = 1.10 mm^−1^
*T* = 295 K
0.33 × 0.21 × 0.18 mm

#### Data collection

Enraf–Nonius CAD-4 diffractometer
7056 measured reflections
6632 independent reflections
5226 reflections with *I* > 2σ(*I*)
*R*_int_ = 0.017
3 standard reflections every 60 min  intensity decay: none

#### Refinement

*R*[*F* ^2^ > 2σ(*F* ^2^)] = 0.043
*wR*(*F* ^2^) = 0.128
*S* = 1.05
6632 reflections
440 parameters
H atoms treated by a mixture of independent and constrained refinement
Δρ_max_ = 0.81 e Å^−3^
Δρ_min_ = −0.58 e Å^−3^

### 

Data collection: *CAD-4 Software* (Enraf–Nonius, 1989 ▶); cell refinement: *CAD-4 Software*; data reduction: *XCAD4* (Harms & Wocadlo, 1995 ▶); program(s) used to solve structure: *SHELXS97* (Sheldrick, 2008 ▶); program(s) used to refine structure: *SHELXL97* (Sheldrick, 2008 ▶); molecular graphics: *ORTEP-3* (Farrugia, 1997 ▶); software used to prepare material for publication: *WinGX* (Farrugia, 1999 ▶), *PLATON* (Spek, 2009 ▶) and *PARST* (Nardelli, 1995 ▶).

## Supplementary Material

Crystal structure: contains datablocks I, global. DOI: 10.1107/S160053681000615X/rk2191sup1.cif

Structure factors: contains datablocks I. DOI: 10.1107/S160053681000615X/rk2191Isup2.hkl

Additional supplementary materials:  crystallographic information; 3D view; checkCIF report

## Figures and Tables

**Table 1:** Hydrogen-bond geometry (Å, °)

*D*—H⋯*A*	*D*—H	H⋯*A*	*D*⋯*A*	*D*—H⋯*A*
O4*A*—H4*A*⋯N1*A*	0.84 (5)	1.82 (5)	2.592 (4)	153 (5)
O5*A*—H5*A*⋯Cl2^i^	0.83 (5)	2.29 (5)	3.094 (4)	167 (3)
O6*A*—H6*A*⋯O5*A*	0.82 (7)	2.30 (6)	2.693 (4)	110 (5)
O6*A*—H6*A*⋯Cl4^i^	0.82 (7)	2.64 (6)	3.317 (3)	141 (5)
O4*B*—H4*B*⋯N1*B*	0.80 (4)	1.86 (4)	2.599 (4)	155 (4)
O5*B*—H5*B*⋯O4*B*	0.77 (6)	2.28 (6)	2.721 (4)	118 (5)
O5*B*—H5*B*⋯Cl1^ii^	0.77 (6)	2.60 (5)	3.217 (3)	139 (5)
O6*B*—H6*B*⋯O5*B*	0.85 (5)	2.32 (5)	2.728 (4)	110 (4)
O6*B*—H6*B*⋯Cl4^iii^	0.85 (5)	2.59 (5)	3.193 (3)	129 (4)
C10*A*—H10*A*⋯O1*A*	0.96	2.34	2.992 (5)	124
C10*B*—H10*F*⋯O1*B*	0.96	2.33	2.978 (4)	124
C10*B*—H10*E*⋯O4*A*	0.96	2.50	3.404 (5)	157
C10*B*—H10*F*⋯O1*B*	0.96	2.33	2.978 (4)	124
C12*A*—H12*A*⋯O6*A*^i^	0.96	2.60	3.307 (6)	131
C12*A*—H12*B*⋯O1*A*	0.96	2.41	3.043 (7)	123
C12*B*—H12*E*⋯O1*B*	0.96	2.40	3.028 (5)	123
C12*A*—H12*C*⋯Cl1^ii^	0.96	2.83	3.715 (5)	154
O7—H7*O*⋯Cl3^iv^	0.82	2.44	3.251 (4)	169
N2*A*—H2*NA*⋯Cl1	0.77 (4)	2.59 (4)	3.287 (3)	154 (4)
N2*B*—H2*NB*⋯O7	0.78 (4)	2.05 (4)	2.826 (5)	175 (4)

Symmetry codes: (i) 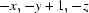; (ii) 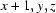; (iii) 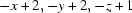; (iv) 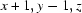.

**Fig. 1.**
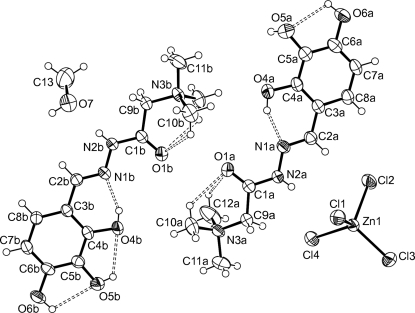
The molecular structure of title compound with atom labels. Displacement ellipsoids are drawn at 50% probability level. H atoms are presented as a small spheres of arbitrary radius. The intramolecular H-bonds are indicated as dashed lines.

**Fig. 2.**
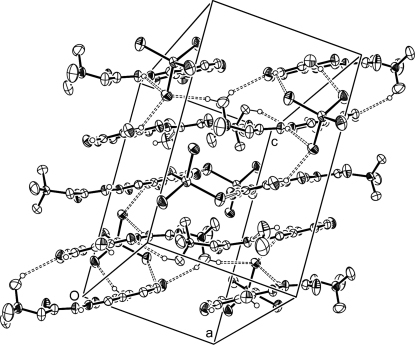
The packing diagram of title compound shows the intermolecular H-bonds (dashed lines). H atoms not involved in intermolecular H-bonds are omitted for clarity.

## supplementary crystallographic information

### Comment

The Schiff base derivatives of Girard reagent are recently investigated as ligands in coordination chemistry (Vojinović *et al.*, 2004 and references therein; Leovac *et al.*, 2006, 2007; Revenko *et al.*, 2009; Vojinović-Ješić *et al.*, 2008, 2010). Considering the specific distribution of charge and simultaneous presence of several donor and acceptor atoms these compounds are able to build extensive hydrogen bonding networks consisting of various types of interactions.

The asymmetric unit of the title compound is given in Fig. 1. Two crystallographically independent cations (A and B) display very similar geometry. The bond lengths and angles within the aliphatic parts of the cations are consistent with those of two previously reported Girard-T based hydrazones (Leovac *et al.*, 2007; Revenko *et al.*, 2009).

Excluding the quaternary ammonium groups, non-hydrogen atoms of two cations (A and B) lie in plane, i.e. the root-mean-square deviations of fitted atoms 0.1Å or less. These approximately planar forms of the molecules are stabilized by number of intramolecular hydrogen bonds with the shortest being O4—H4···N1 (see Table 1). The C9—N3 bonds are single and allow for free rotation of the N(CH_3_)_3_) moiety (as was observed in complexes (Leovac *et al.*, 2007), yet in each of the cations the deviation of the quaternary ammonium groups is restrained due to intramolecular C—H···O interactions.

Anion (ZnCl_4_)^2-^ exhibits regular tetrahedral geometry with Zn—Cl distances comparable to similar anions (Jin *et al.*, 2005; Valkonen *et al.*, 2006). Through O—H···Cl, N—H···Cl and C—H···Cl hydrogen bonds the anion intermediates between five different cations. The strongest among these interactions is rather short and directional O5A—H5A···Cl2^i^ (symmetry code: (i) -*x*, -*y*+1, -*z*) (see Table 1). It is worth noticing that the hydroxyl hydrogen involved in this interaction is the only one (from the six in totals) which significantly deviates from the trihydroxybenzyl moiety.

In the crystal packing, centrosymmetrically related cationic molecules, by C—H···O interactions, with H···O distances all shorter than 2.7Å, associate into corresponding AA and BB dimers. The distances between the parallel planes (passing trough all atoms except N(CH_3_)_3_) are 3.386 (8) and 3.194 (7)Å for AA and BB dimers respectively. The pairs of AA and BB dimers further arrange in approximately parallel fashion (angle between planes of A and B molecule is 0.92 (8)°) along the *c* axis with the closest distance between non-H atoms of 3.407 (5)Å (Fig. 2.). The C—H···O hydrogen interactions relating the dimers mainly include the methyl groups from the quaternary ammonium fragment and oxygen O6 which is in *para*-position concerning the aliphatic fragment.

### Experimental

To a warm solution of *L* (*L* = [(CH_3_)_3_NCH_2_C(O)NHNCHC_6_H_2_(OH)_3_]^+^ Cl^-^) (0.15 g, 0.5 mmol) in *Me*OH (5 ml) was added a solution ZnCl_2_ (anhydrous) (0.07 g, 0.5 mmol) in *Me*OH (3 ml). The reaction mixture was refluxed for 45 min. After two days the resulting light-green crystals have been filtered and washed with methanol and ether (yield 37%).

### Refinement

The H atoms bonded to O atoms of trihydroxybenzyl groups and H atoms bonded to N2 atoms (cations A and B) were located in difference map and refined isotropically. C-bound H atoms were placed in calculated positions (C—H 0.93Å, 0.96Å & 0.97Å) and refined as riding, with *U*_iso_(H) = 1.2(or 1.5)*U*_eq_(C). The refinement of the methanol H resulted in unrealistic positional and thermal parameters, therefore the position of this atom was determined geometrically - O7—H7=0.82Å and *U*_iso_(H7) equal to 1.5*U*_eq_(O7).

### Crystal data

**Table:**

(C_12_H_18_N_3_O_4_)_2_[ZnCl_4_]·CH_4_O	*Z* = 2
*M**_r_* = 775.82	*F*(000) = 804
Triclinic, *P*1	*D*_x_ = 1.523 Mg m^−^^3^
Hall symbol: -P 1	Mo *K*α radiation, λ = 0.71073 Å
*a* = 9.471 (3) Å	Cell parameters from 25 reflections
*b* = 13.389 (4) Å	θ = 11.5–19.4°
*c* = 14.986 (5) Å	µ = 1.10 mm^−^^1^
α = 110.90 (4)°	*T* = 295 K
β = 94.91 (4)°	Prism, light-green
γ = 103.94 (5)°	0.33 × 0.21 × 0.18 mm
*V* = 1691.9 (12) Å^3^	

### Data collection

**Table:**

Enraf–Nonius CAD-4 diffractometer	*R*_int_ = 0.017
Radiation source: fine-focus sealed tube	θ_max_ = 26.0°, θ_min_ = 1.5°
graphite	*h* = 0→11
ω/2θ–scans	*k* = −16→16
7056 measured reflections	*l* = −18→18
6632 independent reflections	3 standard reflections every 60 min
5226 reflections with *I* > 2σ(*I*)	intensity decay: none

### Refinement

**Table:**

Refinement on *F*^2^	Primary atom site location: structure-invariant direct methods
Least-squares matrix: full	Secondary atom site location: difference Fourier map
*R*[*F*^2^ > 2σ(*F*^2^)] = 0.043	Hydrogen site location: inferred from neighbouring sites
*wR*(*F*^2^) = 0.128	H atoms treated by a mixture of independent and constrained refinement
*S* = 1.05	*w* = 1/[σ^2^(*F*_o_^2^) + (0.0754*P*)^2^ + 0.7588*P*] where *P* = (*F*_o_^2^ + 2*F*_c_^2^)/3
6632 reflections	(Δ/σ)_max_ = 0.001
440 parameters	Δρ_max_ = 0.81 e Å^−^^3^
0 restraints	Δρ_min_ = −0.58 e Å^−^^3^

### Special details

**Table:**

Geometry. All s.u.'s (except the s.u. in the dihedral angle between two l.s. planes) are estimated using the full covariance matrix. The cell s.u.'s are taken into account individually in the estimation of s.u.'s in distances, angles and torsion angles; correlations between s.u.'s in cell parameters are only used when they are defined by crystal symmetry. An approximate (isotropic) treatment of cell s.u.'s is used for estimating s.u.'s involving l.s. planes.
Refinement. Refinement of *F*^2^ against ALL reflections. The weighted *R*-factor wR and goodness of fit *S* are based on *F*^2^, conventional *R*-factors *R* are based on *F*, with *F* set to zero for negative *F*^2^. The threshold expression of *F*^2^ > σ(*F*^2^) is used only for calculating *R*-factors(gt) etc. and is not relevant to the choice of reflections for refinement. *R*-factors based on *F*^2^ are statistically about twice as large as those based on *F*, and *R*-factors based on ALL data will be even larger.

### Fractional atomic coordinates and isotropic or equivalent isotropic displacement parameters (Å^2^)

**Table:**

	*x*	*y*	*z*	*U*_iso_*/*U*_eq_	
Zn1	0.12662 (4)	0.97209 (3)	0.27361 (2)	0.03615 (12)	
Cl1	0.08234 (9)	0.87169 (6)	0.36739 (6)	0.0457 (2)	
Cl2	−0.02229 (10)	0.86775 (8)	0.12557 (6)	0.0582 (2)	
Cl3	0.07728 (10)	1.13575 (7)	0.34252 (7)	0.0550 (2)	
Cl4	0.36959 (9)	0.99260 (7)	0.25977 (7)	0.0542 (2)	
O1A	0.3976 (3)	0.5823 (2)	0.2618 (2)	0.0630 (7)	
O4A	0.0152 (3)	0.3662 (2)	0.11211 (17)	0.0436 (5)	
O5A	−0.2207 (3)	0.1888 (2)	−0.00766 (19)	0.0516 (6)	
O6A	−0.4465 (3)	0.2247 (2)	−0.10178 (19)	0.0547 (6)	
O1B	0.6208 (3)	0.44499 (19)	0.23238 (19)	0.0510 (6)	
O4B	0.9987 (2)	0.63770 (19)	0.40319 (17)	0.0425 (5)	
O5B	1.2379 (3)	0.80292 (19)	0.52686 (19)	0.0508 (6)	
O6B	1.4654 (3)	0.7511 (2)	0.60703 (17)	0.0484 (6)	
N1A	0.1257 (3)	0.5816 (2)	0.19456 (18)	0.0365 (5)	
N2A	0.2344 (3)	0.6776 (2)	0.25321 (19)	0.0382 (6)	
N3A	0.6305 (3)	0.7890 (2)	0.36455 (18)	0.0427 (6)	
N1B	0.8729 (2)	0.4242 (2)	0.31181 (16)	0.0327 (5)	
N2B	0.7550 (3)	0.3346 (2)	0.25584 (18)	0.0336 (5)	
N3B	0.3590 (3)	0.2561 (2)	0.14515 (17)	0.0354 (5)	
C1A	0.3662 (3)	0.6692 (3)	0.2858 (2)	0.0390 (6)	
C2A	0.0097 (3)	0.5939 (3)	0.1539 (2)	0.0365 (6)	
H2A	0.0015	0.6651	0.1655	0.044*	
C3A	−0.1076 (3)	0.4967 (2)	0.0901 (2)	0.0340 (6)	
C4A	−0.1013 (3)	0.3874 (3)	0.0709 (2)	0.0338 (6)	
C5A	−0.2159 (3)	0.2969 (3)	0.0080 (2)	0.0373 (6)	
C6A	−0.3357 (3)	0.3144 (3)	−0.0383 (2)	0.0402 (7)	
C7A	−0.3442 (3)	0.4209 (3)	−0.0204 (2)	0.0424 (7)	
H7A	−0.4253	0.4316	−0.0513	0.051*	
C8A	−0.2319 (3)	0.5109 (3)	0.0430 (2)	0.0395 (7)	
H9A	−0.2381	0.5827	0.0553	0.047*	
C9A	0.4695 (3)	0.7812 (3)	0.3546 (3)	0.0446 (7)	
H9A1	0.4536	0.8381	0.3324	0.054*	
H9A2	0.4432	0.7981	0.4184	0.054*	
C10A	0.6698 (4)	0.7172 (4)	0.4147 (3)	0.0652 (11)	
H10B	0.7738	0.7245	0.4196	0.098*	
H10A	0.6142	0.6405	0.3777	0.098*	
H10C	0.6465	0.7408	0.4786	0.098*	
C11A	0.7131 (5)	0.9079 (3)	0.4276 (4)	0.0787 (14)	
H11A	0.6905	0.9560	0.3975	0.118*	
H11C	0.8177	0.9167	0.4358	0.118*	
H11B	0.6841	0.9272	0.4900	0.118*	
C12A	0.6778 (5)	0.7560 (5)	0.2689 (3)	0.0796 (14)	
H12C	0.7816	0.7622	0.2784	0.119*	
H12A	0.6598	0.8042	0.2377	0.119*	
H12B	0.6226	0.6801	0.2288	0.119*	
C1B	0.6326 (3)	0.3538 (2)	0.2203 (2)	0.0338 (6)	
C2B	0.9820 (3)	0.4038 (2)	0.3508 (2)	0.0329 (6)	
H2B	0.9809	0.3303	0.3378	0.039*	
C3B	1.1081 (3)	0.4953 (2)	0.41539 (19)	0.0307 (6)	
C4B	1.1095 (3)	0.6063 (2)	0.4389 (2)	0.0319 (6)	
C5B	1.2296 (3)	0.6925 (2)	0.5024 (2)	0.0346 (6)	
C6B	1.3468 (3)	0.6685 (2)	0.54445 (19)	0.0347 (6)	
C7B	1.3446 (3)	0.5582 (3)	0.5228 (2)	0.0377 (6)	
H7B	1.4223	0.5420	0.5516	0.045*	
C8B	1.2270 (3)	0.4731 (2)	0.4586 (2)	0.0356 (6)	
H8B	1.2266	0.3994	0.4437	0.043*	
C9B	0.5121 (3)	0.2445 (2)	0.1623 (2)	0.0368 (6)	
H9B2	0.5382	0.2095	0.0997	0.044*	
H9B1	0.5101	0.1948	0.1963	0.044*	
C10B	0.3479 (4)	0.3179 (3)	0.0803 (2)	0.0470 (8)	
H10D	0.3735	0.2794	0.0193	0.071*	
H10F	0.4148	0.3921	0.1108	0.071*	
H10E	0.2484	0.3218	0.0693	0.071*	
C11B	0.2541 (4)	0.1409 (3)	0.0978 (3)	0.0578 (9)	
H11D	0.2795	0.1022	0.0368	0.087*	
H11E	0.1549	0.1453	0.0868	0.087*	
H11F	0.2607	0.1012	0.1394	0.087*	
C12B	0.3156 (4)	0.3150 (3)	0.2399 (2)	0.0504 (8)	
H12D	0.2158	0.3178	0.2276	0.076*	
H12E	0.3813	0.3896	0.2711	0.076*	
H12F	0.3221	0.2751	0.2813	0.076*	
H4B	0.940 (4)	0.579 (3)	0.370 (3)	0.051 (11)*	
H5B	1.166 (6)	0.804 (4)	0.500 (4)	0.085 (17)*	
H6B	1.454 (5)	0.816 (4)	0.621 (3)	0.075 (15)*	
H4A	0.075 (5)	0.430 (4)	0.143 (3)	0.070 (14)*	
H5A	−0.147 (5)	0.177 (4)	−0.030 (3)	0.067 (14)*	
H6A	−0.427 (6)	0.165 (5)	−0.114 (4)	0.10 (2)*	
H2NB	0.758 (4)	0.276 (3)	0.254 (2)	0.038 (9)*	
H2NA	0.212 (4)	0.731 (3)	0.267 (3)	0.044 (11)*	
C13	0.7289 (7)	0.0438 (5)	0.1584 (4)	0.1002 (18)	
H13A	0.8041	0.0717	0.1271	0.150*	
H13B	0.6329	0.0310	0.1224	0.150*	
H13C	0.7380	−0.0251	0.1608	0.150*	
O7	0.7452 (4)	0.1197 (3)	0.2501 (3)	0.0843 (10)	
H7O	0.8265	0.1296	0.2811	0.126*	

### Atomic displacement parameters (Å^2^)

**Table:**

	*U*^11^	*U*^22^	*U*^33^	*U*^12^	*U*^13^	*U*^23^
Zn1	0.03453 (19)	0.03438 (19)	0.0375 (2)	0.00982 (14)	−0.00078 (14)	0.01356 (14)
Cl1	0.0518 (4)	0.0427 (4)	0.0458 (4)	0.0134 (3)	0.0046 (3)	0.0223 (3)
Cl2	0.0511 (5)	0.0706 (6)	0.0384 (4)	0.0232 (4)	−0.0089 (4)	0.0048 (4)
Cl3	0.0607 (5)	0.0396 (4)	0.0602 (5)	0.0222 (4)	−0.0016 (4)	0.0122 (4)
Cl4	0.0351 (4)	0.0513 (5)	0.0666 (5)	0.0088 (3)	0.0055 (4)	0.0155 (4)
O1A	0.0412 (13)	0.0405 (13)	0.0897 (19)	0.0182 (11)	−0.0098 (13)	0.0062 (13)
O4A	0.0375 (11)	0.0466 (13)	0.0444 (12)	0.0177 (10)	−0.0002 (10)	0.0134 (10)
O5A	0.0532 (15)	0.0411 (13)	0.0540 (14)	0.0108 (11)	0.0069 (12)	0.0139 (11)
O6A	0.0368 (12)	0.0543 (16)	0.0525 (14)	0.0054 (11)	−0.0063 (10)	0.0058 (12)
O1B	0.0411 (12)	0.0402 (12)	0.0673 (15)	0.0134 (10)	−0.0047 (11)	0.0181 (11)
O4B	0.0375 (12)	0.0382 (12)	0.0512 (13)	0.0121 (10)	−0.0059 (10)	0.0194 (11)
O5B	0.0522 (14)	0.0348 (12)	0.0558 (15)	0.0079 (10)	−0.0105 (12)	0.0146 (10)
O6B	0.0395 (12)	0.0476 (14)	0.0423 (12)	0.0020 (10)	−0.0094 (10)	0.0104 (11)
O7	0.076 (2)	0.0651 (19)	0.097 (2)	0.0241 (16)	−0.0165 (17)	0.0207 (17)
N1A	0.0286 (12)	0.0391 (13)	0.0355 (13)	0.0067 (10)	0.0029 (10)	0.0103 (10)
N2A	0.0276 (12)	0.0362 (14)	0.0430 (14)	0.0110 (11)	−0.0006 (10)	0.0071 (11)
N3A	0.0303 (13)	0.0478 (15)	0.0376 (13)	0.0064 (11)	−0.0032 (10)	0.0081 (11)
N1B	0.0280 (11)	0.0372 (12)	0.0299 (11)	0.0070 (10)	0.0008 (9)	0.0123 (10)
N2B	0.0293 (12)	0.0325 (13)	0.0351 (12)	0.0083 (10)	−0.0018 (10)	0.0109 (10)
N3B	0.0281 (11)	0.0474 (14)	0.0318 (12)	0.0092 (10)	0.0002 (9)	0.0192 (11)
C1A	0.0309 (14)	0.0390 (16)	0.0417 (16)	0.0099 (12)	0.0029 (12)	0.0108 (13)
C2A	0.0346 (15)	0.0411 (16)	0.0329 (14)	0.0120 (12)	0.0046 (12)	0.0131 (12)
C3A	0.0288 (13)	0.0432 (16)	0.0303 (14)	0.0108 (12)	0.0056 (11)	0.0147 (12)
C4A	0.0286 (13)	0.0459 (16)	0.0292 (13)	0.0141 (12)	0.0068 (11)	0.0149 (12)
C5A	0.0355 (15)	0.0454 (17)	0.0315 (14)	0.0123 (13)	0.0098 (12)	0.0146 (13)
C6A	0.0302 (14)	0.0538 (18)	0.0302 (14)	0.0070 (13)	0.0049 (11)	0.0129 (13)
C7A	0.0305 (15)	0.061 (2)	0.0365 (15)	0.0159 (14)	0.0002 (12)	0.0190 (14)
C8A	0.0364 (15)	0.0487 (17)	0.0365 (15)	0.0168 (13)	0.0044 (12)	0.0177 (13)
C9A	0.0315 (15)	0.0401 (16)	0.0526 (19)	0.0124 (13)	−0.0024 (13)	0.0081 (14)
C10A	0.054 (2)	0.078 (3)	0.064 (2)	0.029 (2)	−0.0094 (18)	0.027 (2)
C11A	0.046 (2)	0.054 (2)	0.096 (3)	−0.0037 (18)	−0.015 (2)	0.003 (2)
C12A	0.048 (2)	0.129 (4)	0.041 (2)	0.008 (2)	0.0077 (17)	0.019 (2)
C1B	0.0295 (13)	0.0395 (16)	0.0304 (13)	0.0096 (12)	0.0018 (11)	0.0126 (12)
C2B	0.0329 (14)	0.0347 (14)	0.0302 (14)	0.0123 (11)	0.0061 (11)	0.0101 (11)
C3B	0.0273 (13)	0.0377 (14)	0.0278 (13)	0.0101 (11)	0.0057 (10)	0.0131 (11)
C4B	0.0277 (13)	0.0418 (15)	0.0299 (13)	0.0107 (11)	0.0049 (11)	0.0182 (12)
C5B	0.0359 (15)	0.0365 (15)	0.0304 (14)	0.0079 (12)	0.0036 (11)	0.0143 (12)
C6B	0.0306 (14)	0.0435 (16)	0.0238 (13)	0.0063 (12)	0.0030 (11)	0.0096 (12)
C7B	0.0307 (14)	0.0494 (17)	0.0338 (14)	0.0165 (13)	0.0029 (11)	0.0148 (13)
C8B	0.0365 (15)	0.0371 (15)	0.0343 (14)	0.0156 (12)	0.0045 (12)	0.0126 (12)
C9B	0.0318 (14)	0.0387 (15)	0.0370 (15)	0.0123 (12)	−0.0016 (12)	0.0121 (12)
C10B	0.0419 (17)	0.068 (2)	0.0420 (17)	0.0188 (16)	0.0038 (14)	0.0324 (16)
C11B	0.0422 (18)	0.058 (2)	0.058 (2)	−0.0027 (16)	−0.0138 (16)	0.0210 (18)
C12B	0.0444 (18)	0.073 (2)	0.0389 (17)	0.0214 (17)	0.0149 (14)	0.0230 (16)
C13	0.108 (4)	0.094 (4)	0.089 (4)	0.060 (3)	0.002 (3)	0.009 (3)

### Geometric parameters (Å, °)

**Table:**

Zn1—Cl3	2.2467 (13)	O4B—C4B	1.357 (3)
Zn1—Cl1	2.2661 (11)	O4B—H4B	0.80 (4)
Zn1—Cl2	2.2731 (18)	O5B—C5B	1.371 (4)
Zn1—Cl4	2.2870 (12)	O5B—H5B	0.77 (5)
O1A—C1A	1.208 (4)	O6B—C6B	1.359 (4)
O4A—C4A	1.356 (3)	O6B—H6B	0.85 (5)
O4A—H4A	0.84 (4)	N1B—C2B	1.276 (4)
O5A—C5A	1.369 (4)	N1B—N2B	1.366 (3)
O5A—H5A	0.82 (5)	N2B—C1B	1.352 (4)
O6A—C6A	1.362 (4)	N2B—H2NB	0.79 (3)
O6A—H6A	0.82 (6)	N3B—C11B	1.493 (4)
N1A—C2A	1.285 (4)	N3B—C10B	1.495 (4)
N1A—N2A	1.373 (4)	N3B—C12B	1.504 (4)
N2A—C1A	1.346 (4)	N3B—C9B	1.506 (4)
N2A—H2NA	0.75 (4)	C1B—C9B	1.523 (4)
N3A—C12A	1.484 (5)	C2B—C3B	1.454 (4)
N3A—C9A	1.494 (4)	C2B—H2B	0.9300
N3A—C11A	1.497 (5)	C3B—C4B	1.396 (4)
N3A—C10A	1.505 (5)	C3B—C8B	1.397 (4)
C1A—C9A	1.513 (4)	C4B—C5B	1.390 (4)
C2A—C3A	1.441 (4)	C5B—C6B	1.389 (4)
C2A—H2A	0.9300	C6B—C7B	1.388 (4)
C3A—C4A	1.405 (4)	C7B—C8B	1.373 (4)
C3A—C8A	1.407 (4)	C7B—H7B	0.9300
C4A—C5A	1.382 (4)	C8B—H8B	0.9300
C5A—C6A	1.390 (4)	C9B—H9B2	0.9700
C6A—C7A	1.378 (5)	C9B—H9B1	0.9700
C7A—C8A	1.371 (5)	C10B—H10D	0.9600
C7A—H7A	0.9300	C10B—H10F	0.9600
C8A—H9A	0.9300	C10B—H10E	0.9600
C9A—H9A1	0.9700	C11B—H11D	0.9600
C9A—H9A2	0.9700	C11B—H11E	0.9600
C10A—H10B	0.9600	C11B—H11F	0.9600
C10A—H10A	0.9600	C12B—H12D	0.9600
C10A—H10C	0.9600	C12B—H12E	0.9600
C11A—H11A	0.9600	C12B—H12F	0.9600
C11A—H11C	0.9600	C13—O7	1.355 (6)
C11A—H11B	0.9600	O7—H7O	0.8200
C12A—H12C	0.9600	C13—H13A	0.9600
C12A—H12A	0.9600	C13—H13B	0.9600
C12A—H12B	0.9600	C13—H13C	0.9600
O1B—C1B	1.203 (4)		
			
Cl3—Zn1—Cl1	109.97 (5)	H10A—C10A—H10C	109.5
Cl3—Zn1—Cl2	110.89 (5)	N3A—C11A—H11A	109.5
Cl1—Zn1—Cl2	107.16 (5)	N3A—C11A—H11C	109.5
Cl3—Zn1—Cl4	113.17 (6)	H11A—C11A—H11C	109.5
Cl1—Zn1—Cl4	105.36 (5)	N3A—C11A—H11B	109.5
Cl2—Zn1—Cl4	109.97 (6)	H11A—C11A—H11B	109.5
C4A—O4A—H4A	103 (3)	H11C—C11A—H11B	109.5
C5A—O5A—H5A	108 (3)	N3A—C12A—H12C	109.5
C6A—O6A—H6A	113 (4)	N3A—C12A—H12A	109.5
C4B—O4B—H4B	102 (3)	H12C—C12A—H12A	109.5
C5B—O5B—H5B	106 (4)	N3A—C12A—H12B	109.5
C6B—O6B—H6B	112 (3)	H12C—C12A—H12B	109.5
C13—O7—H7O	109.5	H12A—C12A—H12B	109.5
C2A—N1A—N2A	116.7 (3)	O1B—C1B—N2B	124.5 (3)
C1A—N2A—N1A	118.6 (3)	O1B—C1B—C9B	124.6 (3)
C1A—N2A—H2NA	126 (3)	N2B—C1B—C9B	110.9 (2)
N1A—N2A—H2NA	116 (3)	N1B—C2B—C3B	120.2 (3)
C12A—N3A—C9A	112.0 (3)	N1B—C2B—H2B	119.9
C12A—N3A—C11A	110.4 (3)	C3B—C2B—H2B	119.9
C9A—N3A—C11A	106.7 (3)	C4B—C3B—C8B	118.9 (3)
C12A—N3A—C10A	108.2 (3)	C4B—C3B—C2B	120.9 (2)
C9A—N3A—C10A	112.0 (3)	C8B—C3B—C2B	120.2 (3)
C11A—N3A—C10A	107.4 (3)	O4B—C4B—C5B	116.0 (3)
C2B—N1B—N2B	117.1 (2)	O4B—C4B—C3B	124.0 (3)
C1B—N2B—N1B	118.6 (3)	C5B—C4B—C3B	120.0 (3)
C1B—N2B—H2NB	124 (2)	O5B—C5B—C6B	117.4 (3)
N1B—N2B—H2NB	116 (2)	O5B—C5B—C4B	122.5 (3)
C11B—N3B—C10B	109.3 (2)	C6B—C5B—C4B	120.1 (3)
C11B—N3B—C12B	108.3 (3)	O6B—C6B—C7B	118.6 (3)
C10B—N3B—C12B	109.5 (3)	O6B—C6B—C5B	121.4 (3)
C11B—N3B—C9B	107.3 (2)	C7B—C6B—C5B	120.1 (3)
C10B—N3B—C9B	111.6 (2)	C8B—C7B—C6B	119.7 (3)
C12B—N3B—C9B	110.8 (2)	C8B—C7B—H7B	120.1
O1A—C1A—N2A	123.6 (3)	C6B—C7B—H7B	120.1
O1A—C1A—C9A	124.5 (3)	C7B—C8B—C3B	121.2 (3)
N2A—C1A—C9A	112.0 (3)	C7B—C8B—H8B	119.4
N1A—C2A—C3A	119.6 (3)	C3B—C8B—H8B	119.4
N1A—C2A—H2A	120.2	N3B—C9B—C1B	114.8 (2)
C3A—C2A—H2A	120.2	N3B—C9B—H9B2	108.6
C4A—C3A—C8A	118.3 (3)	C1B—C9B—H9B2	108.6
C4A—C3A—C2A	122.4 (3)	N3B—C9B—H9B1	108.6
C8A—C3A—C2A	119.2 (3)	C1B—C9B—H9B1	108.6
O4A—C4A—C5A	117.7 (3)	H9B2—C9B—H9B1	107.5
O4A—C4A—C3A	122.1 (3)	N3B—C10B—H10D	109.5
C5A—C4A—C3A	120.2 (3)	N3B—C10B—H10F	109.5
O5A—C5A—C4A	122.5 (3)	H10D—C10B—H10F	109.5
O5A—C5A—C6A	117.6 (3)	N3B—C10B—H10E	109.5
C4A—C5A—C6A	119.8 (3)	H10D—C10B—H10E	109.5
O6A—C6A—C7A	119.7 (3)	H10F—C10B—H10E	109.5
O6A—C6A—C5A	119.4 (3)	N3B—C11B—H11D	109.5
C7A—C6A—C5A	121.0 (3)	N3B—C11B—H11E	109.5
C8A—C7A—C6A	119.5 (3)	H11D—C11B—H11E	109.5
C8A—C7A—H7A	120.3	N3B—C11B—H11F	109.5
C6A—C7A—H7A	120.3	H11D—C11B—H11F	109.5
C7A—C8A—C3A	121.3 (3)	H11E—C11B—H11F	109.5
C7A—C8A—H9A	119.4	N3B—C12B—H12D	109.5
C3A—C8A—H9A	119.4	N3B—C12B—H12E	109.5
N3A—C9A—C1A	115.2 (3)	H12D—C12B—H12E	109.5
N3A—C9A—H9A1	108.5	N3B—C12B—H12F	109.5
C1A—C9A—H9A1	108.5	H12D—C12B—H12F	109.5
N3A—C9A—H9A2	108.5	H12E—C12B—H12F	109.5
C1A—C9A—H9A2	108.5	O7—C13—H13A	109.5
H9A1—C9A—H9A2	107.5	O7—C13—H13B	109.5
N3A—C10A—H10B	109.5	H13A—C13—H13B	109.5
N3A—C10A—H10A	109.5	O7—C13—H13C	109.5
H10B—C10A—H10A	109.5	H13A—C13—H13C	109.5
N3A—C10A—H10C	109.5	H13B—C13—H13C	109.5
H10B—C10A—H10C	109.5		

### Hydrogen-bond geometry (Å, °)

**Table:**

*D*—H···*A*	*D*—H	H···*A*	*D*···*A*	*D*—H···*A*
O4A—H4A···N1A	0.84 (5)	1.82 (5)	2.592 (4)	153 (5)
O5A—H5A···Cl2^i^	0.83 (5)	2.29 (5)	3.094 (4)	167 (3)
O6A—H6A···O5A	0.82 (7)	2.30 (6)	2.693 (4)	110 (5)
O6A—H6A···Cl4^i^	0.82 (7)	2.64 (6)	3.317 (3)	141 (5)
O4B—H4B···N1B	0.80 (4)	1.86 (4)	2.599 (4)	155 (4)
O5B—H5B···O4B	0.77 (6)	2.28 (6)	2.721 (4)	118 (5)
O5B—H5B···Cl1^ii^	0.77 (6)	2.60 (5)	3.217 (3)	139 (5)
O6B—H6B···O5B	0.85 (5)	2.32 (5)	2.728 (4)	110 (4)
O6B—H6B···Cl4^iii^	0.85 (5)	2.59 (5)	3.193 (3)	129 (4)
C10A—H10A···O1A	0.96	2.34	2.992 (5)	124
C10B—H10F···O1B	0.96	2.33	2.978 (4)	124
C10B—H10E···O4A	0.96	2.50	3.404 (5)	157
C10B—H10F···O1B	0.96	2.33	2.978 (4)	124
C12A—H12A···O6A^i^	0.96	2.60	3.307 (6)	131
C12A—H12B···O1A	0.96	2.41	3.043 (7)	123
C12B—H12E···O1B	0.96	2.40	3.028 (5)	123
C12A—H12C···Cl1^ii^	0.96	2.83	3.715 (5)	154
O7—H7O···Cl3^iv^	0.82	2.44	3.251 (4)	169
N2A—H2NA···Cl1	0.77 (4)	2.59 (4)	3.287 (3)	154 (4)
N2B—H2NB···O7	0.78 (4)	2.05 (4)	2.826 (5)	175 (4)

Symmetry codes:  (i) −*x*, −*y*+1, −*z*; (ii) *x*+1, *y*, *z*; (iii) −*x*+2, −*y*+2, −*z*+1; (iv) *x*+1, *y*−1, *z*.
